# The Side Population in Human Lung Cancer Cell Line NCI-H460 Is Enriched in Stem-Like Cancer Cells

**DOI:** 10.1371/journal.pone.0033358

**Published:** 2012-03-13

**Authors:** Yang Shi, Xuelian Fu, Yong Hua, Yang Han, Ying Lu, Junchen Wang

**Affiliations:** 1 Department of Pathology, East Hospital, Tongji University, Shanghai, China; 2 Central Laboratory, East Hospital, Tongji University, Shanghai, China; 3 Research Center for Translational Medicine, Cancer Stem Cell Institute, East Hospital, Tongji University, Shanghai, China; The University of Texas M.D Anderson Cancer Center, United States of America

## Abstract

Lung cancer is among the most lethal malignancies with a high metastasis and recurrence rate. Recent studies indicate that tumors contain a subset of stem-like cancer cells that possess certain stem cell properties. Herein, we used Hoechst 33342 dye efflux assay and flow cytometry to isolate and characterize the side population (SP) cells from human lung cancer cell line NCI-H460 (H460). We show that the H460 SP cells harbor stem-like cells as they can readily form anchorage-independent floating spheres, possess great proliferative potential, and exhibit enhanced tumorigenicity. Importantly, the H460 SP cells were able to self-renew both in vitro and in vivo. Finally, we show that the H460 SP cells preferentially express ABCG2 as well as SMO, a critical mediator of the Hedgehog (HH) signaling, which seems to play an important role in H460 lung cancer cells as its blockage using Cyclopamine greatly inhibits cell-cycle progression. Collectively, our results lend further support to the existence of lung cancer stem cells and also implicate HH signaling in regulating large-cell lung cancer (stem) cells.

## Introduction

It has long been appreciated that most tumors are heterogeneous containing a spectrum of phenotypically different cell types. Work in the past decade indicates that various human solid tumors also contain functionally divergent tumor cells with subpopulations possesing high tumorigenic potential and being able to reconstitute the phenotypic and histologic heterogeneity of the parent tumor when transplanted in immunodeficient mice. Such subsets of tumor cells that possess enhanced tumorigenic capacity have been operationally called tumor-initiating cells or cancer stem cells (CSC), which have now been reported in most solid tumors [1], [2]. Most CSCs have been identified, enriched, and purified using either cell surface marker(s), among which CD44 and CD133 are the most popular, or functional assays, which include side population (SP) [3]–[6] and Aldeflour assays [7], [8]. The SP strategy was initially developed to enrich hematopoietic stem cells [3] and is based on the ability of stem cells, which overexpress detoxifying cell surface pumps ABCG2 and MDR1 (i.e., P-glycoprotein), to efficiently efflux the cell-permeable dye Hoechst 33342 and consequently, on dual wavelength FACS plot to present as a Hoechst-negative population on the ‘side’ (or at the tail). The Aldeflour assay, on the other hand, takes advantage of stem cells overexpressing detoxifying enzymes aldehyde dehydrogenases (ALDH) [7], [8] and therefore, the CSC-enriched population can more efficiently metabolize an experimental ALDH substrate to release more fluorophore.

Lung cancer is the most lethal maligancy world-wide. Work in the past several years indicates that both small-cell (SCLC) and non-small cell (NSCLC) lung cancers contain stem-like cancer cells [9]–[29]. As in most other tumors, ‘lung CSCs’ have been enriched and purified using cell surface markers CD44 or CD133 or using the two functional assays mentioned above. These lung CSCs have been demonstrated to possess high clonal, clonogenic, and frequently, tumorigenic potential and to be generally resistant to therapeutic treatments. The lung cancer stem cells have been reported in long-term cultures as well as in xenografts and primary patient tumors. Of interest, a recent study using genetic mouse models of lung cancer shows that lung tumors with different genetic backgrounds have distinct CSC phenotypes [30], raising the possibility that different patient lung tumors may have different CSC phenotypes. Although the SP technique has been employed to demonstrate CSCs in several lung cancer cell lines [10], [11], [13], [25], it is not known whether all patient tumor-derived lung cancer cell lines possess a SP that is enriched in stem-like cancer cells. Here we further address this question by using the human large-cell large carcinoma line NCI-H460 (H460) and our results reveal that H460 cells possess a SP that is enriched in tumor-initiating cells.

## Results and Discussion

### Cultured human lung cancer cell line NCI-H460 has a SP

**Figure 1:**
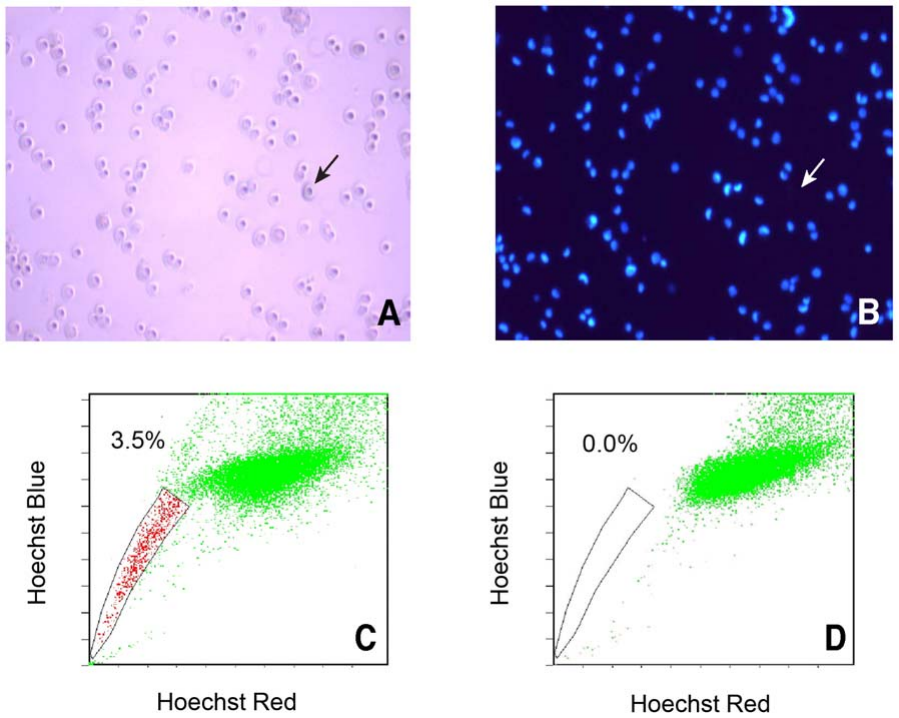
SP analysis in cultured H460 lung cancer cells. A–B, Hoechst staining of H460 cells. Note that although the majority of cells were stained in the nucleus, some cells (indicated by arrows) apparently lacked nuclear Hoechst staining. C–D, SP phenotypes in the absence (C) or presence (D) of verapamil.

We first stained H460 cells with Hoechst 33342, which is actively extruded by verapamil-sensitive ABC transporters in stem cells [3]. When we observed the stained cells under a fluorescence microscope, the majority of nuclei, as expected, appeared blue; however, a small number of nuclei were negative for Hoechst staining (Fig. 1, A and B; the arrows point to a Hoechst-negative cell). We then quantified the SP by dual wavelength flow cytometry [3]–[6], [10], [11], [13], [25]. We detected, in multiple independent H460 cultures, a SP of 3.80±0.5% (n = 9), as illustrated in Fig. 1C. Importantly, the SP was completely eliminated in the presence of verapamil (Fig. 1D), a calcium-channel blocker and a specific inhibitor of ABCG2 and MDR1 used in the clinical treatment of lung cancer [31], indicating the specificity of the SP we detected in H460 cells.

### SP cells demonstrate high proliferative potential and can self-renew

**Figure 2:**
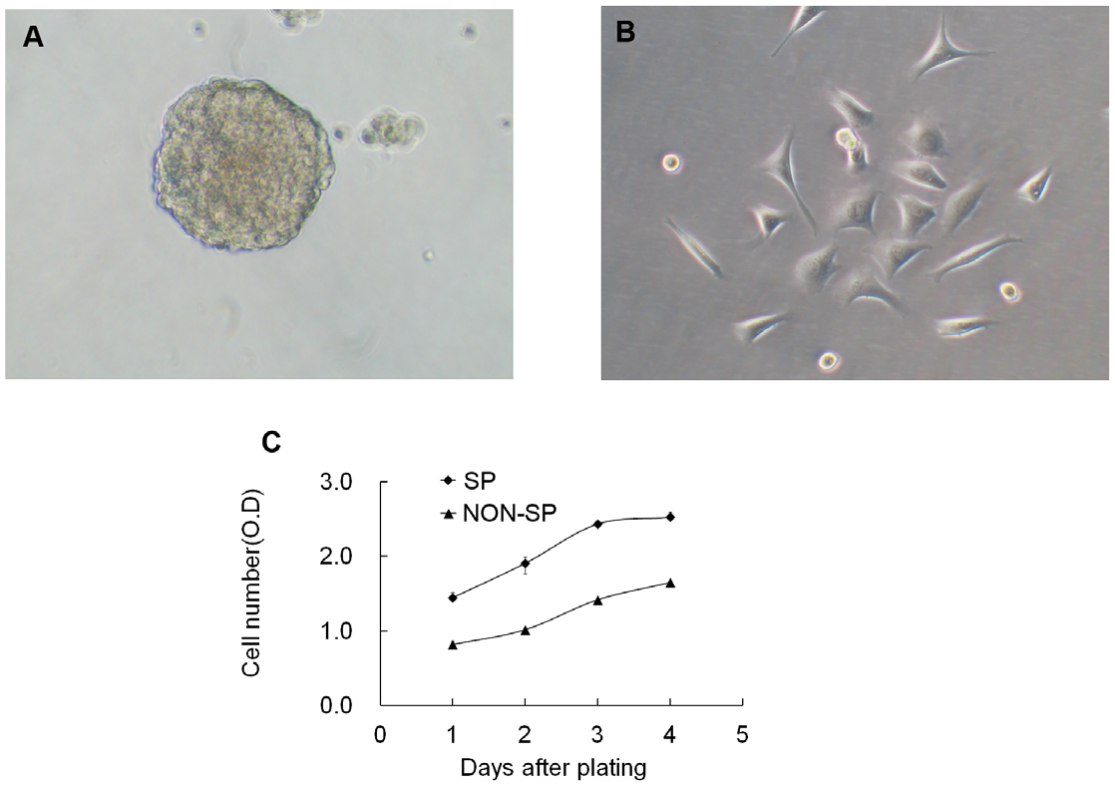
Sphere formation and proliferative capacity of H460 SP and non-SP cells. A–B, Purified SP and non-SP cells were cultured in serum-free medium in anchorage-independent conditions. The SP cells formed typical floating spheres within 4 days (A) whereas the non-SP cells largely established adherent growth (B). C, The SP cells displayed higher proliferative ability than non-SP cells as determined by CCK-8 kit (*P*<0.05 for all time points, Student *t*-test).

**Figure 3:**
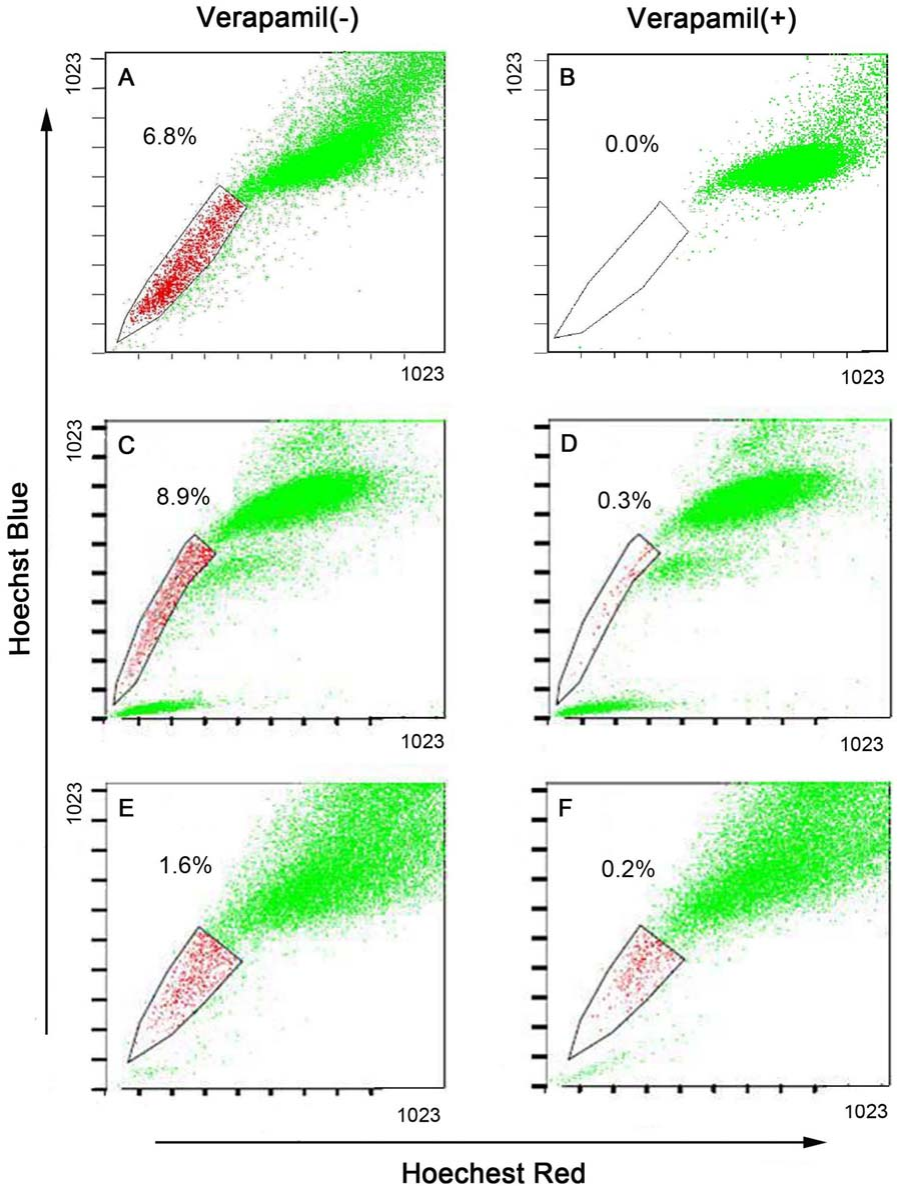
SP re-analysis in various samples. A–B, SP re-analysis when the SP spheres were disaggregated and dissociated cells cultured in normal serum-containing medium for one week. C–D, SP re-analysis in the SP cell-derived tumors. E–F, SP re-analysis in non-SP cell-derived tumors. A, C, E, without verapamil. B, D, F, with verapamil.

Up to now most stem-like cells have been demonstrated to have an ability to form free-floating spheres in anchorage-independent conditions [4]–[6], [10], [11], [13]. Furthermore, CSCs have been reported to possess a high proliferative potential. To determine whether the H460 SP cells have similar CSC-associated properties, we first cultured purified SP and non-SP cells in serum-free condition (see Methods). We observed that the H460 SP cells formed typical floating spheres with an efficiency of 4.8±0.1% (Fig. 2A) whereas the non-SP cells mostly showed adherent growth pattern (Fig. 2B) with much lower sphere-forming capacity (0.8±0.3%). CCK-8 proliferation experiment (see Method) also revealed higher proliferative potential in the SP cells compared to the non-SP cells (Fig. 2C). When the primary SP cell-derived spheres were dissociated and passaged, they readily formed secondary spheres (data not shown). When dissociated primary SP spheres were cultured in complete medium containing fetal bovine serum (FBS) for one week, new SP cells (6.2±0.8%) were detected with the majority cells being non-SP cells (Fig. 3A). Again, the SP phenotype was completely blocked in the presence of verapamil (Fig. 3B). These results suggest that SP cells can self-renew *in vitro*.

### The H460 SP cells demonstrate higher tumorigenicity than corresponding non-SP cells

**Figure 4:**
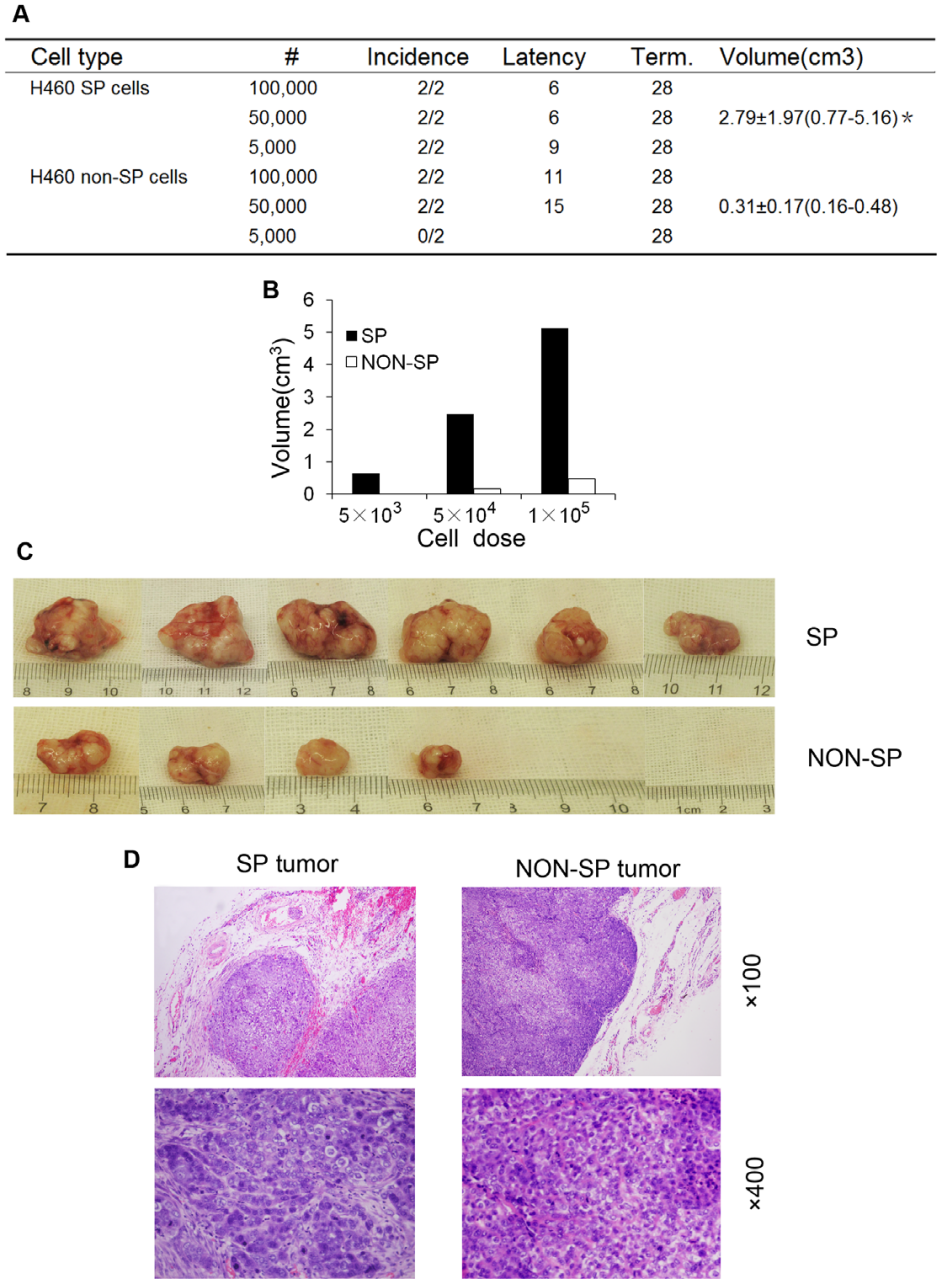
High tumorigenicity in SP cells. A, Table presentation of the tumorigenic potential of H460 SP and non-SP cells. Three parameters of tumorigenicity, i.e., tumor incidence, latency, and volume (*P<0.01) were shown. All animals were terminated (term.) 28 days after implantation. B, The SP cells regenerated larger tumors than corresponding non-SP cells at every cell dose. C, Gross tumor images when tumors were harvested at day 28 after s.c. injection of the SP and non-SP cells into NOD/SCID mice. D, Representative HE-stained photomicrographs of SP and non-SP tumors.

The gold standard for assaying CSC properties is to perform limiting-dilution tumor transplantation experiments [1], [2] and to compare tumorigenicity, commonly measured by tumor incidence, latency (i.e., the time between tumor cell implantation to when tumors can first be palpated), growth rate (i.e., tumor volume), and the endpoint tumor weight. We purified the H460 SP and non-SP cells and injected increasing numbers of cells in Matrigel subcutaneously (s.c) into the nonobese diabetic/severe combined immunodeficiency (NOD/SCID) mice (Fig. 4A). We found that the SP cells overall showed higher tumorigenicity than non-SP cells. For example, the SP cells regenerated tumors at the lowest cell number (i.e., 5,000) implanted whereas the non-SP cells did not regenerate any tumor at this cell dose (Fig. 4A). The SP cells regenerated 6/6 tumors (i.e., 100%) whereas the non-SP cells gave rise to 4/6 tumors (67%; *P* = 0.039, Chi-Square tests). In addition, the SP cells established tumors with a shorter latency than the corresponding non-SP cells (Fig. 4A; *P* = 0.007). Furthermore, the H460 SP cell-derived tumors grew faster leading to much larger tumors than non-SP cell derived tumors (Fig. 4A–C; *P*<0.01). Histologically, the regenerated xenograft tumors had morphological characteristics of the large-cell lung cancer. In particular, the SP tumors displayed abundance of cells and mitotic figures and showed evident capsular invasion (Fig. 4D, left). In contrast, the non-SP tumors exhibited more necrotic areas (Fig. 4D, right).

It was surprising and potentially interesting that the non-SP cells, at 50,000 and 100,000, also regenerated tumors although the tumors were smaller (Fig. 4B–C). Since the non-SP cells did not form tumors at 5,000 cells (Fig. 4A), the easiest explanation would be that the non-SP cell population might be contaminated with a small number of SP cells, which would give rise to tumors when large numbers of non-SP cells were implanted. Alternatively, non-SP cells might be able to ‘de-differentiate’ back to SP cells at a low rate such that in vivo some non-SP cells were converted into SP cells, which then gave rise to tumors. This latter scenario was recently demonstrated by others in several cultured tumor cell systems [32]. To begin exploring these different possibilities, we analyzed the SP composition in both SP and non-SP cell derived H460 tumors. We observed that the SP tumors contained 8.4±0.6% SP cells (Fig. 3C–D) whereas the non-SP tumors contained 1.4±0.2% SP cells (Fig. 3E–F). Although these results could not definitely distinguish contamination from conversion, they did provide an explanation for why the non-SP cells still gave rise to tumors – it was because the non-SP tumors contained SP cells.

### The stem cell markers ABCG2 and SMO are highly expressed in SP cells

**Figure 5:**
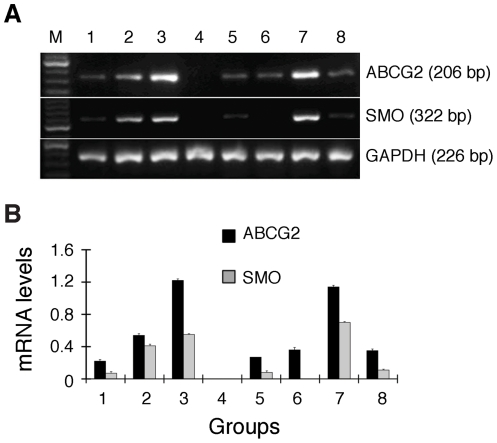
RT-PCR analysis of ABCG2 and SMO mRNA levels. A, Representative RT-PCR gel images. M, marker; lane 1, NCI-H460 cells normally cultured in serum-containing medium; lane 2, the H460 spheres; lane 3, purified SP cells; lane 4, purified non-SP cells; lane 5, the Tomatidine control group; lane 6, the Cyclopamine experimental group; lane 7, SP tumors; lane 8, non-SP tumors. B, Quantitative presentation of ABCG2 and SMO mRNA levels as determined by densitometry (*P*<0.001, except ABCG2 mRNA lane 6 vs. lane 8 and SMO mRNA lane 1 vs. lane 5 and lane 4 vs. lane 6).

The SP phenotype in hematopoietic stem cells is mediated primarily by ABCG2 with some involvement of MDR1 or multi-drug resistance protein 1 [31]. Many CSC-enriched SP's overexpress ABCG2 [4], [6]. We therefore analyzed ABCG2 mRNA levels and observed that, compared to regular FBS-cultured monolayer H460 cells (Fig. 5A, lane 1), both spheres (Fig. 5A, lane 2) and purified SP cells (Fig. 5A, lane 3) expressed significantly increased ABCG2 mRNA levels whereas purified non-SP H460 cells virtually lacked ABCG2 expression (Fig. 5A, lane 4). Likewise, the SP tumors (Fig. 5A, lane 7) also expressed higher levels of ABCG2 than corresponding non-SP tumors (Fig. 5A, lane 8). A quantitative presentation was shown in Fig. 5B.

Tumorigenic lung cancer cells including SP cells have been shown to preferentially express self-renewal molecules such as Oct-4, Nanog, Bmi-1, and c-Kit [14], [16], [17], [22], [29], Notch signaling components [18], [28], and Wnt/β-catenin [23]. Emerging evidence suggests that the Hedgehog (HH) signaling pathway may also be intimately involved in lung cancer development and progression [33], [34]. Activation of HH signaling requires the transmembrane protein Smoothened (SMO) as a critical mediator of the HH signaling [35] and ABCG2 may act in the upstream regulation of the Hh signaling pathway to protect the stemness of the SP compartment [36]. We therefore determined the mRNA levels of SMO in the same set of samples that were used for ABCG2 analysis. Strikingly, very much like ABCG2, the SMO mRNA levels were significantly elevated in H460 spheres, SP cells, and SP cell-derived tumors (Fig. 5A–B). To our knowledge, this finding may represent the very first to link SMO overexpression to CSC-enriched lung cancer cells.

### The SMO inhibitor Cyclopamine inhibits H460 cell proliferation

**Figure 6:**
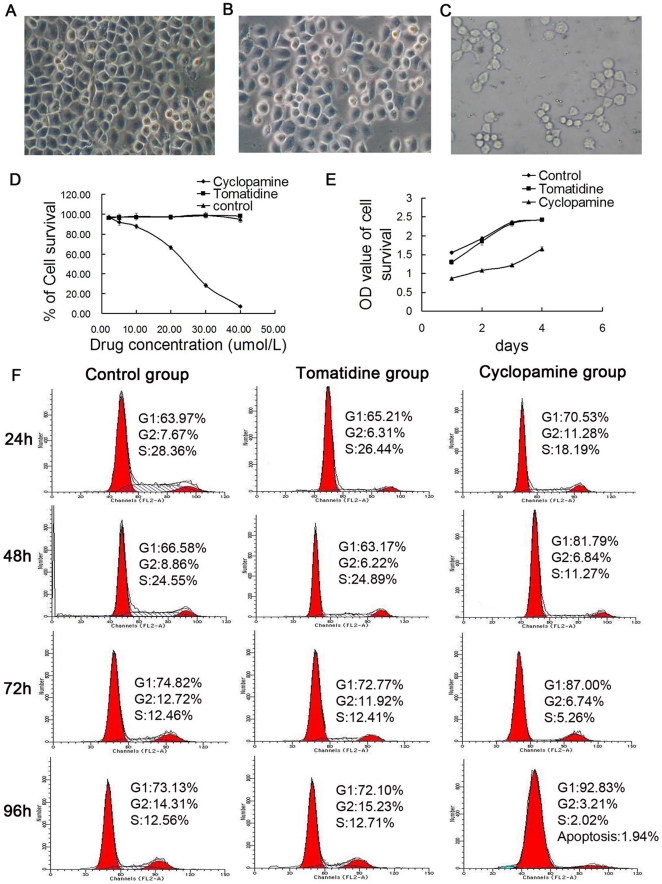
Cyclopamine inhibits H460 cell proliferation. A–C, Representative photomicrographs of H460 cells 72 h after treatment with vehicle control (A), Tomatidine (B), or Cyclopamine (C). Original maginifications: ×200. D, Cyclopamine dose-dependently inhibited H460 cell proliferation (*P*<0.05; one-way ANOVA). E, Time course of Cyclopamine inhibition of H460 cells (*P*<0.05, one-way ANOVA). Cyclopamine was used at 20 µmol/L. F, Effects of Cyclopamine on the cell cycle of H460 cells.

HH signaling pathway has been implicated in the maintenance of stem or progenitor cells in many adult tissues. This pathway regulates cell proliferation, tissue polarity, and cell differentiation during normal development. Importantly, abnormal HH pathway activation, such as high-levels of SMO expression, may play a role in the maintenance of the capacity of tumor stem cells, favoring self-renewal, and proliferation of their progeny [36], [37]. To determine whether HH signaling plays a role in H460 cells, we employed Cyclopamine, a steroidal alkaloid that inhibits SMO activity. As a control for Cyclopamine, we used a structurally related alkaloid, Tomatidine, which does not affect SMO activity and HH signaling. Compared to Tomatidine, Cyclopamine appeared to reduce the low endogenous levels of SMO mRNA (Fig. 5A, lanes 5 and 6). Compared to the vehicle control and Tomatidine group, Cyclopamine dose- and time-dependently inhibited the growth of H460 cells when used at 2–40 µmol/L (Fig. 6A–E). Cell-cycle analysis demonstrated that Cyclopamine, at 20 µmol/L, time-dependently caused H460 cells to arrest in the G1/S phase of the cell cycle (Fig. 6F). Specifically, Cyclopamine treatment increased the G1 cells from ∼71% at 24 h to ∼93% at 96 h whereas decreased S-phase cells from 18% at 24 h to 2% at 96 h (Fig. 6F). At 96 h, slightly increased apoptosis (i.e., ∼2%) was also observed (Fig. 6F). It should be noted that both vehicle and Tomatidine control groups also showed time-dependent increases in G1-phase cells and time-related decreases in S-phase cells (Fig. 6F), likely due to the fact that all cells were cultured for up to 96 h without replenishing media. Hence, exhaustion of growth factors and nutrients caused partial cell-cycle arrest in the control groups. Together, these results demonstrate that the HH signaling is vital in H460 cells and blockade of HH signaling dramatically causes cell-cycle arrest. Since SMO is preferentially expressed in CSC-enriched SP and spheres(Fig. 5), we surmise that the HH signaling imposes its effects likely on the lung CSCs.

In summary, in this study we present evidence that the H460 human large-cell lung carcinoma cell line contains a detectable SP. Further, we show that the H460 SP cells harbor stem-like cells as they can readily form anchorage-independent floating spheres, possess great proliferative potential, and exhibit enhanced tumor-regenerating capacity. Importantly, the H460 SP cells seem to be able to self-renew in vitro (evidenced by replating sphere cells in regular culture medium; Fig. 3A) and in vivo (evidenced by the SP tumors containing a small percentage of SP cells with the majority being non-SP cells; Fig. 3C). Finally, we show that the H460 SP cells preferentially express ABCG2 as well as SMO, a critical mediator of the HH signaling, which seems to play an important role in H460 lung cancer cells as its blockage using Cyclopamine greatly inhibits cell-cycle progression. Collectively, our results provide further support to the presence of stem-like cancer cells in cultured lung cancer cell lines and also implicate HH signaling in regulating large-cell lung cancer (stem) cells.

## Materials and Methods

### Ethics statement

ALL experiments were conducted according to the Institutional Ethical Guidelines. All animal-related studies were approved by the Tongji University Institutional IACUC committee. NOD/SCID mice (the permit number: SYXK20070005) at SPF level were used for tumor cell implantation experiments in this study. All other studies presented herein were investigator-initiated and did not require approval from other regulatory bodies.

### Cell lines and animals

Human large-cell lung carcinoma cell line NCI-H460 was bought from the Shanghai Institutes for Biological Sciences, CAS (Shanghai, China) and maintained in medium recommended by ATCC. All media were supplemented with 1% penicillin/streptomycin and 10% fetal bovine serum (FBS; Invitrogen-Life Technologies). Cells were incubated in a humidified incubator at 37°C supplied with 5% CO2. Cells were routinely maintained in 75-cm^2^ tissue culture flasks (Corning Incorporated, USA) and harvested using 0.25% trypsin when they were in logarithmic phase of growth for SP analysis. The nonobese diabetic/severe combined immunodeficiency (NOD/SCID) mice were purchased from the Shanghai SLAC Laboratory Animal Co. Ltd.

### SP analysis

The basic protocol was based on Goodell et al [3]. Briefly, the NCI-H460 Cells were resuspended at 1×10^6^/mL in pre-warmed DMEM (Invitrogen-Life Technologies). Hoechst 33342 dye was added at a final concentration of 5 µg/mL in the presence or absence of verepamil (50 µmol/L; Sigma) and the cells were incubated at 37°C for 90 min with intermittent shaking. At the end of the incubation, the cells were washed with ice-cold HBSS (Invitrogen-Life Technologies), centrifuged down at 4°C, and resuspended in ice-cold HBSS. Propidium iodide (Sigma) at a final concentration of 2 µg/mL was added to the cells to gate viable cells. The cell preparations were filtered through a 40-µm cell strainer to obtain single cell suspension. Flow cytometric analyses and sorting were performed on a Fluorescence Activated Cell Sorter (FACS, Beckman Coulter Epics Altra).

### Tumor cell implantation experiments

FACS-purified SP and non-SP H460 cells were mixted with Matrigel (Becton Dickinson), and then subcutaneously (s.c) injected into NOD/SCID mice. Groups of mice were inoculated with SP cells or non-SP cells at 5×10^3^, 5×10^4^ and 1×10^5^, respectively. Tumor growth was monitored on weekly basis and individual tumor volumes were measured using a digital caliper and approximated according to the formula V = 1/2ab^2^ (a being the long diameter and b the short diameter of the tumor). At the end of experiments, mice were sacrificed after 4 weeks and tumors harvested, measured, and photographed. The internal organs such as lung and liver were carefully observed for metastasis nodules and tumor sections were scrutinized under the microscope. Finally, tumors were also digested to make single-cell suspension for SP re-analysis.

### Sphere formation assays in serum-free cultures

The SP and non-SP cells were cultured in serum-free DMEM-F12 (Invitrogen-Life Technologies), supplemented with 20 ng/mL epidermal growth factor (EGF), 10 ng/mL basic fibroblast growth factor (bFGF), 5 µg/mL insulin (all from Sigma). Cells (1000/well) were plated in 96-well culture dishes in 200 µL of growth medium and 20 µL of medium per well was added every 2 days. The number of spheres (Φ>150 µm) for each well was evaluated after 5 days of culture.

### RT-PCR analysis

Cells were harvested and total RNA was extracted and prepared for RT-PCR by using a PrimeScript™ RT-PCR Kit (Takara, Kyoto, Japan). Cycle parameters for ABCG2, SMO, and GAPDH cDNAs were 30 sec at 94°C, 30 sec at 58°C (for ABCG2 and GAPDH) or 55°C (for SMO), and 45 sec at 72°C for 35 cycles, respectively. The primers for RT–PCR were as follows: ABCG2 (F) 5′-CACCTTATTGGCCTCAGGAA-3′, ABCG2 (R) 5′-CCTGCTTGGAAGGCTCTATG-3′, SMO (F) 5′-TTACCTTCAGCTGCCACTTCTACG-3′, SMO (R) 5′-GCCTTGGCAATCATCTTGCTCTTC-3′, GAPDH (F) 5′-ACGACCACTTTGTCAAGCTC-3′, and GAPDH (R) 5′-GGTCTACATGGCAACTGTGA-3′.

### Cell proliferation Assay

Cell proliferation assays were performed using the Cell Counting Kit-8 (CCK-8, Dojindo, Kumamoto, Japan). Cells were plated in 96-well plates at 5×10^4^ cells per well and cultured in the growth medium. Cyclopamine and Tomatidine (all from Sigma) were added, respectively, at a concentration of 20 µmol/L and appropriate growth medium was added in control samples. CCK-8 was added in each well at 24, 48, 72 and 96 h, and, after an additional 4-h culture, the absorbance of each well was determined and growth curve was plotted. The cell-cycle profiles were analyzed by flow cytometry.

### Statistical analysis

Data were generally presented as the mean ± S.D and statistical differences between experimental groups were analyzed by Student's *t-*test, one-way ANOVA, or linear regression using statistical software SPSS11.5 and according to the nature of data analyzed. A *P*<0.05 was considered statistically significant in all cases.
